# Less is not more: 401(k) plan information and retirement planning choices

**DOI:** 10.1017/s1474747221000445

**Published:** 2021-12-07

**Authors:** Eric Cardella, Charlene M. Kalenkoski, Michael Parent

**Affiliations:** 1 Texas Tech University, Lubbock, USA; 2 The University of Texas at Austin, Austin, USA

**Keywords:** Retirement planning, 401(k), information complexity, nudge, choice architecture

## Abstract

This paper presents the results of a choice experiment that is designed to examine whether changing how plan information is presented affects planned retirement-savings behavior. The main hypothesis is that providing plan information in a more concise format with helpful recommendations, rather than providing lengthy and detailed information, will alter retirement-planning choices. The specific choices examined include: whether to enroll, how much to contribute, and how to structure (broadly) the asset allocation. The choice experiment is conducted on three different samples: (i) a Qualtrics panel of new employees, (ii) a Qualtrics panel of job seekers, and (iii) a sample of business-school students. Our results suggest that, controlling for demographic and other factors, our main hypothesis was not supported by the data in any of the samples. Thus, the data cast some doubt on the notion that simplifying and condensing the retirement-plan information presented to employees will result in vastly different retirement-planning choices.

## 1. Introduction

More than half of Americans fail to save enough for retirement. According to Rhee (2013), about 45% of US households lack sufficient retirement savings and, for working households about to retire, the median household’s retirement savings is just $12,000.^1^ Kirkham (2016) similarly shows that about 33% of Americans do not have any retirement savings and, among those near retirement age, about 54% have retirement-savings amounts that fall below the recommended threshold. Thus, many of these individuals will rely on Social Security, a program that is widely viewed as becoming increasingly financially strained. Therefore, it is generally viewed as important to identify possible mechanisms that can help people save more for retirement. This study investigates how changing the way 401(k) plan information is presented can plausibly impact participation and planning behavior.^2^ In particular, it examines whether presenting (hypothetical) plan information in a more concise and tractable manner with salient recommendation affects: (i) anticipated participation rates, (ii) anticipated contribution levels, and (iii) anticipated asset-allocation decisions.

There are many reasons why Americans are not saving enough for retirement or are doing so (potentially) sub-optimally (Agnew, 2006; Tang *et al*., 2010; Choi *et al*., 2011). One important *potential* explanation is the complexity of the decision-making process (Campbell, 2006; Goldin *et al*., 2020), especially given the excessive paperwork and limited time typically available to make retirement-planning decisions (Madrian and Shea, 2001; Iyengar *et al*., 2004; Beshears *et al*., 2013). In addition, if employees are offered defined-contribution plans, which have become increasingly more likely and now outnumber defined-benefit plans (Campbell *et al*., 2011), they must decide how much to contribute and in what financial products to invest. This process can be quite challenging and overwhelming, which might cause individuals to procrastinate in their enrollment decision (O’Donoghue and Rabin, 1998; Madrian and Shea, 2001; Carroll *et al*., 2009; Beshears *et al*., 2013), fail to enroll (Goldin *et al*., 2020), or make inefficient portfolio decisions (Tang *et al*., 2010). Moreover, employees often will resort to using ‘rules of thumb’ or rely on the choices or advice of others in making their retirement decisions (Benartzi and Thaler, 2007), and often will stick with default options (e.g., contribution rates and asset allocations) set by the employer, even if it is not optimal for the individual (Choi *et al*., 2006; Beshears *et al*., 2013; Goda and Manchester, 2013); thus, tending to follow the ‘path of least resistance’ (Choi *et al*., 2006).

A possible avenue through which retirement-planning choices might be impacted is the manner in which information is provided (e.g., Tversky and Kahneman, 1981; Kahneman and Tversky, 2000). Specifically, simplifying the enrollment information, which typically is very lengthy and complex (Beshears *et al*., 2013; Clark *et al*., 2014; Bateman *et al*., 2016a, 2016b).^3^ This type of information intervention would fall broadly under the categorization of being a ‘nudge’ (Thaler and Sunstein, 2008).^4^ In contrast to traditional economic levers such as taxes, subsidies, and regulation, nudges alter the choice setting in a way that can predictably impact behavior (e.g., changing the default options), while still preserving the choice set and underlying economic incentives (see Hummel and Maedche, 2019 for a review of efficacy of nudges across multiple domains). Because simplicity is widely considered a major component of successful nudges (Sunstein, 2013; Halpern, 2015), the focus of this study is to consider the extent to which presenting plan information in a simplified way with salient recommendations can impact critical retirement-planning decisions – enrollment, contribution level, and portfolio allocation.^5^

To do so, we conduct a hypothetical choice experiment administered via survey using a Qualtrics Panel of recently hired employees.^6^ For robustness, we utilize two additional samples: a Qualtrics Panel of full-time job seekers, and a sample of business-school students. Respondents were provided with information about a hypothetical, but realistic, employer-sponsored 401(k) plan. We randomly varied whether respondents received plan information in the form of: (i) a convoluted, *long* description with all information written out in lengthy paragraphs, or (ii) a compact, *short* description with information presented in a concise table and prominently displayed ‘useful guidelines’ about retirement planning. Respondents were then asked a series of questions related to their anticipated behavior, based on the provided plan information.

The main hypothesis is that presenting the plan information in a more simplified and tractable manner would impact anticipated retirement-planning choices – enrollment, contribution level, and portfolio allocation. Moreover, because the simplified version of the plan information provides recommendations (e.g., rules of thumb) in a more salient manner, we also examine whether choices are more in line with these specific recommendations: (i) increased anticipated participation rates, (ii) increased anticipated contribution rates, and (iii) an anticipated portfolio allocation that reflects a suggested, age-based distribution between stock and bonds. All these outcomes are *generally* viewed as improved retirement planning outcomes (e.g., Bell, March 21, 2017; https://www.bankrate.com/retirement/8-rules-of-thumb-on-saving-and-retirement/).

After controlling for demographic and other factors, no significant differences were found between the control group that received the long version and the treatment group that received the short version in terms of their anticipated enrollment rates, confidence in their enrollment decisions, anticipated contribution rates, or their anticipated portfolio allocations. These results were robust across the two additional samples we consider. Additional data analysis related to the plan manipulation revealed that respondents, on average, spent more time viewing the long description but tended to process the plan information with a similar degree of accuracy. Overall, we find essentially no effect of providing simplified information on respondent’s anticipated retirement-planning behaviors. Thus, our results cast some doubt on the idea that providing plan information in a more concise and tractable way will significantly alter retirement-planning behaviors. However, this could be viewed in a more positive light in that the simplified information will be more accessible and easier to process for employees, which can save time, while generally resulting in similar retirement-planning outcomes. In essence, our results indicate that there is (plausibly) no downside to providing simplified plan information. It is worth noting that the one factor that was consistently associated with anticipated behaviors is financial literacy, suggesting that educational initiatives might be more effective than manipulating plan presentation *if* the goal is to promote the following retirement planning outcomes – increased participation, increased contribution rates (at least up to taking full advantage of employer-matching), and more prudent, age-based asset allocation.

## 2. Relevant literature

In this section, we first briefly discuss the prior literature related to how information presentation and complexity specifically impacts retirement planning behaviors.^7^ We then provide a more comprehensive review of the prior literature focusing on retirement-savings decisions and the factors that influence plan-participation behaviors.

### 2.1 Information presentation and complexity on retirement planning behaviors

Most closely related to the focus of this study, there are a few papers that investigate how information presentation and complexity impact retirement-planning decisions. Beshears *et al*. (2013) argue that the complexity associated with plan-enrollment decisions can lead to procrastination and a failure to enroll. They examine the impact of a ‘Quick Enrollment’ option that allows individuals to enroll with a pre-selected contribution rate and asset allocation. They find that reducing the complexity of the problem through quick enrollment increases plan participation by about 10–20 percentage points. Moreover, there is strong persistence of the pre-selected option with significantly more people contributing at the pre-specified level (either 2% or 4%) and maintaining the pre-specified asset allocation. However, these interventions reduce the choice set of individuals and the pre-selected contribution rate and asset allocation may not be optimal for all employees. While Beshears *et al*. (2013) consider a very extreme case of simplification, our study considers a subtler manipulation where the information people receive is simplified but they do not face a restricted choice environment.

Clark *et al*. (2014) use a field experiment to examine how *nudging* employees through a follow-up flyer impacts plan enrollment. In particular, they consider a manipulation where non-participating employees at a large institution receive a simplified flyer with information about the benefits of participating in the plan and the importance of saving for retirement. They find that providing this simplified and encouraging information did increase participation, especially for males and younger, lower-income employees. Dolls *et al*. (2018) document a similar effect that providing information (by letter) about the expected returns in the German pension system increases private retirement savings. Goldin *et al*. (2020) show that sending an email describing how to enroll improves plan enrollment and also that suggesting a specific contribution rate even further improves participation. These prior studies consider how sending a (simplified) message can increase participation. Our study contributes to this line of research by examining how *simplifying* the presentation of the plan information impacts plan participation, while holding constant the content of the information that is provided across both the treatment and control conditions; namely, all people got a message, and what varied was the complexity of the message. In addition, we also examine other decisions, including planned contribution percentage and planned asset allocation.

Agnew and Szykman (2005) show that increased complexity can result in information overload, especially for those who are less financially literate, which can result in an increased propensity to stick with defaults. Kaufmann and Weber (2013) find that more aggregation in presentation of investment returns results in greater risk-taking behavior, suggesting that providing investors with more aggregated information on returns results in more optimal investment choices. Tse *et al*. (2016) find that more complex advertised fee structures result in more decision errors and higher likelihood of sticking with the default in a stylized, portfolio-choice experiment. Thus, reducing complexity (and the choice set) appears to improve financial decision making.

Relatedly, several papers have shown that the manner by which information is presented can impact retirement savings behaviors (e.g., Bernatzi and Thaler, 1999; Bateman *et al*., 2014, 2015, 2016a; McGowan and Lunn, 2020), as well as what specific information is included in plan disclosures (e.g., Bateman *et al*., 2016b). Regarding the presentation of information, McGowan and Lunn (2020) use a controlled experiment and show that presenting information on the link between contribution levels and expected retirement outcomes in diagram form (compared to a table) impacts planned behavior; specifically, people are more likely to give advice to increase retirement contributions after seeing the diagram. In a series of choice-experiments, Bateman *et al*. (2014, 2015, 2016a) show that the manner by which portfolio risk is conveyed can impact planned portfolio decisions.^8^

If suboptimal retirement savings behavior is a result, at least in part, of the complexity associated with the choice environment, then it is important to better understand how streamlining the decision process could possibly lead to improved retirement savings choices. With this aim in mind, our study focuses specifically on streamlining the way that 401(k) plan information is provided to potential participants. In this regard, our current study focuses on the question of *how* information is provided (holding constant the content of the information) – presenting information in a more concise manner – while much of the prior literature focuses on the questions of *what* information is provided.

### 2.2 Factors impacting enrollment decisions

One important set of factors shown to influence 401(k) enrollment is employee characteristics. In particular, age is associated positively with participation rates (Bassett *et al*., 1998; Madrian and Shea, 2001; Munnell *et al*., 2001). This may be because younger individuals may not yet have access to jobs that offer 401(k) plans, still may be obtaining an education, or are just stepping onto the career ladder. A similar explanation may be used with respect to the level of education as greater education improves access to a 401(k) plan (Brown and Weisbenner, 2014). Another factor that also is associated with enrollment decisions in 401(k) plans is income (Bassett *et al*., 1998; Madrian and Shea, 2001; Brown and Weisbenner, 2014). People with low incomes, especially youth, may be overwhelmed with debt payments and other budgetary constraints such that they feel that they cannot participate in 401(k) plans (Clark *et al*., 2014). Longer job tenure also has been found to result in a higher enrollment in 401(k) plans (Bassett *et al*., 1998; Madrian and Shea, 2001; Clark *et al*., 2014). Individuals’ financial knowledge also correlates positively with enrollment in a 401(k) plan (Lusardi and Mitchell, 2011; Brown and Weisbenner, 2014).^9^,^10^ Finally, an individual’s planning horizon may be an important factor in explaining participation rates in 401(k) plans, with having a shorter planning horizon being associated with lower participation (Munnell *et al*., 2001).

Many factors related to specific plan attributes also have been shown to be important determinants in retirement-plan-enrollment decisions. Notably, employer-matching funds provide an incentive to enroll in 401(k) plans, and have been shown to stimulate enrollment (e.g., Papke and Poterba, 1995; Bassett *et al*., 1998; Hansen, 1999; Huberman *et al*., 2007; Micthell *et al*., 2007; Clark *et al*., 2014). However, after a certain level, participation rates increase at a decreasing rate as the employer match increases. Besides plan features, peer effects also are believed to influence enrollment decisions in 401(k) plans. Duflo and Saez (2002) find that employees are more likely to enroll in a retirement plan when their colleagues are enrolled. Thus, participation rates in 401(k) plans are likely to increase in environments where existing employees largely participate in 401(k) plans.

Prior research also suggests that automatic enrollment into a plan (as the default) is very effective at increasing enrollment. Madrian and Shea (2001) find that, when a 401(k) plan is set as the default option, enrollment numbers significantly increase in the plan by 86%, from a participation rate of 49% prior to its institution. Similarly, Choi *et al*. (2004) extend Madrian and Shea’s (2001) research and find that automatic enrollment increases participation by 85%. Relatedly, Carroll *et al*. (2009) show that the more subtle intervention of requiring new employees to make an ‘active’ choice also results in a sizable increase in 401(k) plan participation.

### 2.3 Factors impacting contribution decisions

Employees who enroll in 401(k) plans also are expected to choose their contribution rates. In addition to increasing participation rates, default options have been shown to affect contribution rates. Madrian and Shea (2001) and Choi *et al*. (2004) report that many new plan participants tend to maintain the default saving rates associated with automatic enrollment. For instance, Madrian and Shea find that 61% of automatically enrolled employees in their study continued to maintain the default rate, likely because some participants take the default rate to be investment advice. The authors explain that, because the default savings rates are low, this phenomenon tends to affect the retirement wealth of participants negatively, which can then result in insufficient retirement income (Benartzi and Thaler, 2007). Thaler and Benartzi (2004) propose a novel solution to this problem – the ‘Save More Tomorrow’ program – which essentially defaults people into pre-committing to allocating a portion of future raises into their retirement plan. Importantly, the authors find that participation in the program successfully increases savings rates from 3.5% to 13.6% by the 4th pay raise, compared to a 6% rate for those who did not participate.

The availability of an employer match also is expected to motivate 401(k) plan participants to increase their saving rates. For example, Munnell *et al*. (2001) show that the availability of a match increases contribution rates by 0.7 percentage points. However, the effect of match size on contribution rates diminishes over time. In addition to the presence of an employer match, an employee’s saving in 401(k) plans may be influenced by the availability of borrowing opportunities. Munnell *et al*. (2001) show that the ability of participants to borrow from their 401(k) contributions, which is valuable particularly for liquidity-constrained employees, positively influences contribution rates by 2.6 percentage points.

In terms of employee characteristics, Clark *et al*. (2014), Huberman *et al*. (2007), and Stawski *et al*. (2007) all suggest that contribution rates increase with age. This may be because younger individuals have more debt and/or less goal clarity than older adults. Gender also is found to be a factor determining 401(k) contribution rates. The contribution rates for men exceed those of women (Madrian and Shea, 2001; Clark *et al*., 2014). Huberman *et al*. (2007) suggest that this could be because, on average, males earn higher incomes than females.

### 2.4 Factors impacting asset-allocation decisions

Participants in 401(k) plans typically face the additional decision of how to structure their portfolios. According to Brinson *et al*. (1995), asset allocation accounts for approximately 90% of the variation in security returns. Yet, participants spend very little time choosing their asset allocations. In an experiment involving the University of Southern California’s staff employees, Benartzi and Thaler (1999) find that many respondents spend no more than one hour in making their asset-allocation decisions. In another experiment, Choi *et al*. (2011) find that people take, on average, 36 minutes to make their asset-allocation decisions.

Bernatzi and Thaler (2007) offer a few possible explanations as to why employees do not spend much time making their asset-allocation decisions, given their complexity and importance. First, some 401(k) participants prefer instead to rely on the advice of their peers, friends, and family. Second, some participants simply adopt the default investment option associated with automatic enrollment. Third, people have a naïve view of diversification and thus choose to distribute their funds equally among the investment assets available in their company’s 401(k) plan. Relatedly, Iyengar and Kamenica (2010) show that the number of investment funds offered through a plan impacts asset allocation via the likelihood of contributing to equity funds and the percentage contributed to equity funds.

Automatic enrollment also may affect asset-allocation. Research by Agnew *et al*. (2003) shows the prevalence of inertia in asset-allocation decisions; namely, those who are automatically enrolled tend to invest less in equities because they are more likely to stick with conservative defaults. Madrian and Shea (2001) and Choi *et al*. (2004) provide further evidence to buttress this finding. Choi *et al*. (2004) also find that as job tenure increases, the number of participants choosing the default declines. Camilleri *et al*. (2019) also find that people are more likely to follow ‘smart’ default allocations that progressively become more conservative in their investment mix as the time window until retirement gets smaller (i.e., the person is closer to retirement).

Lastly, employee attributes also have been shown to shape asset allocations. For example, men are more likely to have stocks in their retirement portfolios and to invest more in stocks than women (Barber and Odean, 2001; Agnew *et al*., 2003). Similarly, Neelakantan (2010) find that men generally exhibit more risk tolerance than women in their retirement accounts. Heo *et al*. (2016) also show that risk preferences mediate the relation between gender and investment behavior. According to Agnew *et al*. (2003), married individuals also tend to allocate more funds to equities than single adults.^11^ Clark *et al*. (2017) document that people with more financial knowledge hold a higher proportion of stocks in their portfolio. Lastly, Choi *et al*. (2004) show that low-income participants are more likely to stick with a money-market-default allocation, while Agnew *et al*. (2003) find that participants in high-income brackets invest a greater proportion of their 401(k) wealth in equities.

## 3 Experimental design

An online choice experiment was conducted to identify how the presentation of information about a hypothetical retirement-savings plan impacts anticipated savings choices. Respondents were presented with realistic retirement-savings-plan information. We systematically manipulated whether the plan information was presented in either a *short*, concise format or a *long* format.

### 3.1 Survey procedure

Respondents first were asked a series of general demographic questions, followed by five general financial literacy questions (Lusardi and Mitchell, 2011).^12^ The respondents then were presented with the retirement-savings scenario, followed by some detailed hypothetical information about the plan (discussed in the next section). Respondents then were asked the following questions about their hypothetical, anticipated participation behavior based on the plan information provided:^13^

1. Would you enroll in the plan?
2. How confident are you in your enrollment decision?
3. What percentage of your salary would you contribute to the plan (or stick with the default)?
4. How would you plan on investing your contribution (stick with the default allocation or choose your own allocation)?
5. What percentages of your contribution would you invest in stocks versus bonds?

We then elicited self-reported information about the actual retirement-planning experience of respondents. This information includes: (i) the age they plan to retire; (ii) whether and what type of plan is offered by their current employer; (iii) whether they participate in their current employer’s sponsored plan if one is offered; and (iv) how much they currently are saving for retirement.

### 3.2 Manipulation of the presentation of the plan

The goal of the experimental scenario was to provide plan information that reasonably approximated both the content and the detail that employees might actually receive.^14^ Respondents were given information relating to the following broad categories: overview of the plan, the enrollment process, investment options, level of employer matching, vesting, distributions from the plan, tax benefits, and a general disclaimer about risks and other things to consider. The main manipulation consisted of two conditions: (i) long version *(Long)* and (ii) short version *(Short)*. A full copy of each of these versions and the information that was provided about the plan is presented in the Supplemental Appendix.

In the *Long* version, the plan information for each of the categories was written out in full paragraph form (roughly a paragraph for each category). In total, the plan description in the *Long* version contained 894 words, 5,343 total characters, and was two pages. Alternatively, in the *Short* version, the information was presented in a much simpler and concise way; respondents were given a table highlighting the key information for each category of plan information. Importantly, the plan description in the *Short* version contained 331 words, 1,989 total characters, and was less than one standard page. Moreover, in the *Short* version, respondents were also presented with some key information in the form of *useful guidelines* that had suggested actions, which were intended to ‘help with their enrollment and contribution decisions’. Importantly, these statements were also provided in the *Long* version, albeit *buried* within the written-out text. The following questions and answers were provided in the *Short* version:

**Question: Should you enroll?** Answer: Yes, it is generally regarded as a good idea to contribute to an employer-sponsored, 401(k) plan when there is matching.

**Question: How much should you invest?** Answer: In order to take full advantage of the employer match, you should contribute 4%, but a general rule of thumb is that you should be saving at least 10% of your income toward retirement.

**Question: How should you invest your contributions?** Answer: A general rule of thumb is that the percentage invested in bonds should equal your age.

### 3.3 Sample selection of full-time new employees

To ensure that the sample of respondents was representative of the type of person that likely would make retirement-planning decisions, the following inclusion criteria were imposed: (i) current employment, (ii) started their job within the last year, and (iii) annual income over $50,000. The ex-post questionnaire revealed that approximately 87% of respondents reported that their employer offered a retirement savings plan, and 73% of all respondents reported that they participated in their employer’s plan (i.e., 83% of the 87% who reported a plan was offered).^15^ Participants were recruited through Qualtrics Panels, which is a survey-recruitment platform that draws from a diverse, national pool of registered users. In this regard, we view this sample as being generally more representative than other possible types of samples (e.g., a regional sample, a firm-specific sample, or a student sample). After completing the survey, the participants were compensated directly by Qualtrics. In total, the sample consists of 600 respondents. Data were collected in the fall of 2017, and the average time to complete the survey was just over 6 minutes.^16^

## 4. Results

Table 1 shows descriptive statistics for the socioeconomic variables for the entire sample of 600 new employees, and separately for the *Short* condition (*N* = 302) and the *Long* condition (*N* = 298). Importantly, there are no significant differences across the two conditions (at the 5% level), which is expected given randomization to condition; thus, differences in the measured anticipated choices can be attributed causally to exposure to the experimental condition. One important thing to note is that a very high percentage, 90%, said they anticipated enrolling in the hypothetical 401(k) plan.^17^ While this is higher than the unconditional average empirical enrollment rates in such plans, it is not much higher than the empirical averages reported for comparable individuals with relatively high levels of education and annual income levels over $50,000 per year – 78–85% (Brady and Bass, 2019).

### 4.1 Enrollment

The first decision for participants was actively choosing whether they anticipate: (1) enrolling in the 401(k) plan, (2) not enrolling in the 401(k) plan, or (3) waiting to enroll at a later time. From Table 2 we see that 88% of respondents who received the *Short* version selected choice (1) that they would enroll in the plan, while 92% of those who received the *Long* version selected choice (1) that they would enroll. They are marginally statistically different (χ^2^ test: p = 0.084). However, the marginal effects of a probit model presented in the first column of Table 3 show that, after controlling for personal characteristics and other possible explanatory variables, there is no significant difference in the probability of anticipated enrollment in the 401(k) plan across the *Short* and *Long* conditions.^18^

Respondents who were part-time workers in real life had a 0.14 lower probability of anticipated enrollment, even though the hypothetical job was not part-time. Such workers are less likely to enroll in real life and this is affecting their choices in the hypothetical situation as well. Older respondents also are more likely to choose to enroll in the hypothetical plan as they would in real life. This is also true for married respondents. Thus, real-life choices seem to be affecting choices in the hypothetical situation.

As a follow-up to their enrollment decision, we also asked participants to rate their confidence in their decision (0% = not at all confident to 100% = completely confident). We wanted to allow for the possibility that the plan information manipulation could impact their confidence. Specifically, the *Short* form with the salient guidelines might reduce their uncertainty and, hence, make them more confident. From Table 2 we see that participants were, on average, quite confident in their enrollment decision: 78.7% in the *Short* condition and 79.2% in the *Long* condition. This difference is not statistically significant. The regression specification in column 2 of Table 3 confirms no significant effect of the *Short* condition on the reported confidence. Overall, providing brief information and recommendations via the short description of the plan had little impact on respondents’ hypothetical enrollment in the 401(k) plan or their confidence in this decision.^19^

Examining the other controls, *financial literacy* – measured as the number of questions answered correctly on the five questions presented in Footnote 12 – is positively related to confidence; getting an additional question correct translates into over a 3-percentage-point increase in confidence. We also see that part-time workers had lower confidence than full-time workers by almost 7 percentage points, while age and married also were positively related to confidence in the enrollment decision.

### 4.2 Contribution rate

Those respondents who said that they would enroll in the hypothetical plan were then asked what percentage of their salary they anticipated they would contribute. The two options were to: (i) stick with the default contribution level or (ii) write in their desired contribution percentage. The plan information stated that the default contribution level was 4%, the maximum amount that would be fully matched by the employer. It also included a statement that it is a general rule-of-thumb to save 10% of your salary toward retirement.

Table 2 shows that the average anticipated contribution rate was 6.7% in the *Short* condition and 6.4% in the *Long* condition. These are not significantly different (*t*-test: p = 0.655). Column 3 of Table 3 shows the estimated coefficients from an OLS regression of the percentage of earnings contributed to the plan on an indicator for the *Short* condition and other control variables.^20^ The indicator for receiving the *Short* condition is insignificant. These results confirm that receiving the *Short* version did not affect how much people who would enroll would contribute. We also performed some additional analyses on the specific contribution levels that were chosen: (i) the percentage of respondents who stuck with the *default* 4% contribution level, or (ii) the *suggested* 10% level. Those results show that 68% of respondents in the *Short* condition and 69% in the *Long* condition contributed at the 4% level, while only 7.8% of enrollees in the *Short* condition and 6.5% in the *Long* condition contributed at the 10% level. Neither of these differences is statistically significantly different; moreover, we also find no statistically significant differences in the entire distribution of contribution levels across conditions. Overall, providing plan information in the shorter, more concise manner, including a highlighted 10% rule-of-thumb suggestion, seems to have had little impact on the amount contributed. Financial literacy did not appear to have any association with anticipated contribution rates.

### 4.3 Asset allocation

Conditional on choosing to enroll, respondents were asked how they would allocate their contributions between stocks and bonds. Specifically, they had the following two options: (i) stick with the default allocation (50% stocks and 50% bonds), or (ii) write in the percentages they would allocate toward stocks and bonds (with the total between the two being required to sum to 100%).^21^

Table 2 shows that, of those choosing to enroll, 61% chose the default. Of the remaining 39% who didn’t choose the default, the average percentage allocated to stocks was 57.7% in the *Short* condition and 59.3% in the *Long* condition. These are not statistically different from each other (*t*-test: p = 0.548). Furthermore, there are also no statistically significant differences in the distribution of % allocated to stocks across distributions. Lastly, we examine the data concerning the recommendation provided in the *Short* version that the percentage invested in bonds should be equal to a person’s age. We find that virtually none of the respondents followed this recommendation (only five respondents from each condition), and there was no statistically significant difference in this proportion across the *Short* and *Long* conditions.

Column 4 of Table 3 shows the marginal effects of a probit model of choosing the default allocation, if enrolled; the *Short* version condition has no meaningful effect on choosing the default. Column 5 of Table 3 shows the results of an OLS regression of the percent stock allocation, if enrolled and not choosing the default. Again, the *Short* condition has no effect on the anticipated percentage of funds allocated to stocks. However, higher financial literacy is negatively related to the likelihood of choosing the default, as well as positively related to the percentage allocated to stocks, as we might expect. Part-time and younger workers were more likely to choose the default, as expected. Surprisingly, given their greater confidence on average in real life, males were marginally more likely to choose the default in the hypothetical example. None of these employment and demographic variables affected the anticipated percentage of stocks chosen. Overall, providing the specific recommendations described here seems to have had little impact on the percentages of contributions allocated to stocks and bonds.

## 5 Replication using two alternative samples

After documenting that making the plan information more concise had little effect on the anticipated decisions of a Qualtrics panel of recently employed adults across the United States, concerns arose about the *possibility* that respondents’ prior enrollment choices acted as an anchor for their behavior in the survey; these prior choices could have then *crowded-out* the influence of the manipulation of the presentation of the plan information. To mitigate this potential issue and to draw more robust conclusions about the possible impact on enrollment behavior of the way plan information is presented, we replicated the choice experiment using two additional samples. The first was a different Quatrics panel of *job seekers*, who reported they were not employed full-time but were either currently looking or planned to be looking for full-time employment – referred to as the *seeking-employment sample*. The second additional sample was business-school students – we refer to this sample as the *student sample.* The motivation for these additional samples was to capture populations that: (i) presumably were less likely to have been enrolled in an employer-sponsored retirement plan, and (ii) would likely be faced with the opportunity to participate in such an employer-sponsored plan in the near future. Thus, these samples represent the population of *soon-to-be*, full-time employees who will be making retirement-saving decisions regarding employer-sponsored 401(k) plans.

### 5.1 Results from seeking employment Qualtrics Panel

Participants were again recruited through Qualtrics Panels. Except for the different inclusion criteria, all other procedural parts of the study were the same. In total, the sample consists of 420 respondents (split between the two conditions: *n* = 215 for *Long* version and *n* = 205 for *Short* version). Data were collected in spring 2019, and respondents spent roughly 7 minutes, on average, completing the survey. Importantly, only 14% of the sample reported they were currently saving for retirement via an employer-sponsored plan. In terms of age, this sample ranges from 18 to 80 years old with a mean and median of 38.5 and 37 years old, respectively; thus, a diverse set of ages are represented. Table 4 shows the aggregate descriptive statistics for the seeking-employment sample. Importantly, across all the measured socioeconomic characteristics, there is only one significant difference – *part-time worker* – across the *Short* and *Long* conditions within this sample of job seekers; thus, there is sufficient balance across conditions that our randomization into condition appears to be effective.

We again examine the five main planning choices of interest: *enrollment in 401(k) plan*, *confidence in the enrollment decision*, *contribution rate*, choice of *default allocation*, and choice of *asset allocation* if not the default. Table 5 compares the averages of these five variables across the *Short* and *Long* conditions.^22^ Importantly, as with the employed sample, we find little difference in anticipated behaviors across the *Short* and *Long* conditions for the seeking-employment sample. Namely, Table 5 reveals that there are no significant differences at the 5% level in any of the five choice variables across the two conditions. Table 6 replicates the regression analysis from Table 3 using only the seeking-employment sample and, consistent with the unconditional means, reveals that the indicator for *Short* condition is not significant at the 5% level for any of the five models pertaining to the five planning measures. Moreover, we again do not observe any significant differences across conditions in terms of behavior consistent with the specific recommendations – contribution rates of 4% or 10%, or allocation of bonds equal to age. Overall, the data from the full-time job seekers reveal very little impact of receiving the *Short* form on anticipated retirement-plan choices. However, financial literacy is positively associated with anticipated enrollment and confidence in that decision, and negatively associated with the likelihood of choosing the default, as expected. Older respondents are again more confident and less likely to choose the default. Age is also positively correlated with planned contributions such that older respondents have a marginally higher anticipated contribution rate.

### 5.2 Results from the student sample

Students composing this sample were enrolled in the Rawls College of Business at Texas Tech University.^23^ In total, 233 participants from this student database completed the survey. Data were collected in fall 2017, and respondents spent roughly 6 minutes on average completing the survey. Importantly, this sample of students is much different from the Qualtrics panel of new employees that we analyzed first. In particular, only 21% (compared to 34%) reported that they currently save for retirement. Moreover, only 19% (compared to 55%) reported that their current employer offers a retirement-savings plan (401(k) or equivalent) and only 6% (compared to 42%) of the total sample actually participates in an employer-sponsored plan in real life. Table 7 shows the descriptive statistics for the sample of students. There are, again, no statistically significant differences in any of these measures (at the 10% level) across conditions, indicating acceptable randomization.

Table 8 presents descriptive statistics for the same five planning choices. Importantly, the results from the student sample are generally consistent with the results from the two Qualtrics panels. In particular, we see no significant differences in any of the five outcome variables between the *Short* and *Long* conditions. Table 9 shows the effect of being in the *Short* condition on each of these five dependent variables while controlling for other possible explanatory variables. Consistent with the results from comparisons of the unconditional means reported in Table 8, there are no statistically significant effects of being in the *Short* condition on anticipated: enrollment, confidence in the enrollment decision, contribution rate, or self-selected asset-allocation; there is a marginally statistically significant positive effect on choosing the default. Consistent with the prior two samples, we also do not find any significant differences in the propensity to follow the recommendations – 4% or 10% contribution level, and % allocated to bonds equal to age. Lastly, greater financial literacy is positively correlated with greater confidence, a greater percentage of stocks chosen, and an increased contribution rate.

Overall, the fact that we see little impact of the *Short* version on anticipated behavior in a diverse sample of newly hired employees with salaries over $50 K (who presumably have experience with retirement planning), a diverse sample of those seeking employment, and a sample of business students (who presumably have no experience with retirement planning), provides a more robust conclusion about the plausibly limited impact of simplified plan information on anticipated enrollment behavior.

## 6 Evaluation of plan information

Based on the results reported, we provide robust evidence that the *Short* form manipulation of the presentation of the hypothetical 401(k) plan had little impact on anticipated planning behavior. We collected additional data to gain further insights into possible differences in how the two plan-information presentations were perceived. We solicited responses from an additional sample of 196 business school students from the same database that comprised the student sample. Of these, 100 were presented the *Short* version and 96 were presented with the *Long* version. Up through viewing the plan information, the procedure for this sample was identical to the previous samples. Directly after viewing the plan information (but unbeknownst at the time of viewing the plan info), we collected several additional measures related to their comprehension of the plan features, as well as their subjective perceptions regarding the presentation of the plan information. Data were collected in fall 2019, and respondents spent roughly 7 minutes on average completing the survey.

To measure comprehension of the specific plan information, we asked respondents six multiple choice questions testing their knowledge of the plan features that were prominently presented in the plan description. The six questions were the following:

1. How many days do you have to enroll in the plan?
2. Up to what % of your salary will the employer match?
3. What is the general rule of thumb for what percentage you should be contributing?
4. What is the general rule of thumb about what percentage of your portfolio be invested in bonds?
5. If you choose to enroll, but don’t make a contribution decision, what happens?
6. If you enroll, but don’t make any investment decisions, what happens?

The idea with these questions was to get an *objective* measure of how presentation of the plan information impacts respondent’s knowledge regarding the important plan features. To measure subjective perceptions, we asked five additional Likert-scale questions. These questions related to how easy the plan information was to understand, how easy it was to find the information, how comprehensive the information was, how confident they were in making decisions, and whether the information could have been presented in a more simplified manner. Lastly, we also collected data on how long respondents spent viewing the plan information screen.^24^

Regarding the objective comprehension of the plan, the average number of correctly answered questions (out of a total of 6) was 3.59 for the *Long* version and 3.57 for the *Short* version; these are not significantly different from each other. Thus, across both conditions, respondents appeared to accumulate a similar level of knowledge about the plan. In terms of subjective perceptions, the only reported difference was that respondents who viewed the *Long* version were more likely to agree with the statement ‘the plan information could have been presented in a more simplified way’ compared to respondents viewing the *Short* version (p = 0.010). There were no differences in perceptions about ease of understanding the plan, ease of finding information about the plan, how comprehensive the plan information was, or how confident they would be making decisions based on the information.

Lastly, we measured how long each participant spent viewing the plan description. Respondents spent, on average, 56 seconds viewing the *Short* version and nearly three times that long – 152 seconds – viewing the *Long* version, which is significantly different (p = 0.017).^25^ Taken together, these data suggest that respondents in our study spent less time viewing the *Short* version and perceived it to have been presented in a more simplified manner, compared to the *Long* version. Yet, they were able to glean a similar level of information from both plan descriptions and, hence, make similar decisions about anticipated retirement-saving behavior.

## 7. Conclusion

This paper describes the results of a choice experiment using three distinct samples: a Qualtrics Panel of new employees, a Qualtrics Panel of full-time job seekers, and business-school students. Participants in all three samples were provided with either a *long* or *short* description of a hypothetical 401(k) plan, and then asked questions about anticipated retirement-savings behavior. The main hypothesis was that providing abbreviated, concise information with clear recommendations would alter anticipated plan-participation choices. However, controlling for demographic and other factors, this hypothesis was not supported by the data. Namely, we found negligible differences between those who received the long description and those who received the short description in the following self-reported, anticipated outcomes: enrollment rates, contribution levels, and asset allocations. Importantly, this pattern holds in each of the three distinct populations surveyed. To the extent that increased plan participation, increased contribution rates, and asset allocations are more in line with general rules of thumb, which might lead to more desirable outcomes, our results suggest that simplifying the presentation of plan information to employees might not result in *improved* retirement-planning choices.

Additional data collection revealed that respondents did spend significantly less time viewing the simplified plan description. However, respondents demonstrated a similar (objectively measured) degree of comprehension of the plan information across both conditions. Moreover, they (subjectively) perceived the plan descriptions in a similar way, with the exception that respondents rated the short description as being more simplified. This supports the idea that the long description was, indeed, more complex so they spent more time viewing it; but the long description didn’t result in inferior knowledge of the plan and, as a result, did not significantly impact their anticipated retirement-planning decisions.

Overall, the data do not support the idea that presenting 401(k) plan information in a simpler, more compact way (without limiting their choices, as previous studies have done), will alter employees’ retirement-planning choices. On the one hand, our study casts some doubt on the potential effectiveness of implementing policies that would mandate concise and more accessible information disclosures about optional, employee-sponsored retirement plans as a means of improving retirement-planning outcomes. On the other hand, when we view these findings through a broader lens where new employees might be time constrained, our results could have a more positive interpretation. That is, reducing the complexity of retirement-planning information might result in qualitatively similar planning outcomes, while enabling employees to focus more time on other tasks (e.g., satisfying other new-hire demands). One potential caveat is that we didn’t make the plan information simple *enough.* This is a valid criticism, but we would also argue that there are certainly bounds as to how simplified you can present information while still providing employees with the requisite amount of information they need to make an informed decision, and also satisfy any mandatory information disclosure obligations. Swinging the pendulum the other way, it is also possible that the long version of the plan information was not *long enough* to adequately represent real-life plan information. However, given time and feasibility constraints in implementing the experimental study, it was not practical to make the long version much longer. Notwithstanding the differences in the plan description length, the short description also featured guidelines that were displayed in a prominent manner. Overall, what we consider to be a substantially more streamlined plan description with a more salient recommendation did not impact anticipated retirement planning choices in our study.

More generally, our results suggest that subtle or passive manipulations – like changing the way information is presented – are unlikely to result in large behavior changes related to retirement planning. In light of some of the prior literature (e.g., Clark *et al*., 2014; Dolls *et al*., 2018; Goldin *et al*., 2020) pointing toward the effectiveness of reinforcing plan information through follow-up correspondence (flyer, letter, email), our results would indicate that the formulation of the content in these messages is of less importance; rather, what’s important is that they actually get these messages.^26^ Our results seem to also reinforce prior findings that stronger policies – like defaulting opt-in (e.g., Madrian and Shea, 2001; Choi *et al*., 2004), changing the defaults (e.g., Carroll *et al*., 2009; Beshears *et al*., 2013) or creating ‘smart’ defaults (e.g., Thaler and Benartzi, 2004; Camilleri *et al*., 2019) – are likely to be the most effective way to induce behavioral changes (see Hummel and Maedche, 2019 for further discussion on the effectiveness of defaults). Moreover, we did find that financial literacy was positively associated with better choices across all three main samples.^27^ This suggests that increasing financial literacy – possibly through educational programs (as suggested, among others, by Agnew and Szykman, 2005; Clark *et al*., 2006; Tang *et al*., 2010; Lusardi and Mitchell, 2014; Jappelli and Padula, 2015) – would improve retirement-planning decisions and outcomes. Given that so many people choose the default options, it may be most useful to focus on designing defaults in such a way as to improve individuals’ retirement planning outcomes.^28^

## Supplementary Material

Sup material

## Figures and Tables

**Table 1.** Descriptive statistics by condition (new employee sample)

Socioeconomic explanatory variables	Full sample (*n* = 600)	*Short* condition (*n* = 302)	*Long* condition (*n* = 298)	p-value
Financial literacy (# of questions answered correctly; ranges from 0 to 5)	3.31	3.28	3.34	0.661
Part-time worker ( = 1 if yes)	0.09	0.08	0.10	0.386
Age	34.11	33.88	34.34	0.712
Male ( = 1 if male)	0.51	0.48	0.55	0.061
Married ( = 1 if yes)	0.62	0.62	0.61	0.964
Race/ethnicity				
Hispanic	0.21	0.22	0.20	0.690
Native American	0.06	0.05	0.06	0.484
Asian	0.10	0.09	0.10	0.678
Black	0.14	0.14	0.14	0.907
Hawaiian or Pacific Islander	0.02	0.03	0.01	0.262
White	0.75	0.77	0.73	0.302
Income level				
$50,001–$60,000	0.24	0.24	0.25	0.849
$60,001–$70,000	0.14	0.16	0.13	0.416
$70,001–$80,000	0.13	0.12	0.14	0.394
$80,001–$90,000	0.08	0.09	0.08	0.923
$90,001–$100,000	0.08	0.09	0.07	0.293
$100,001–$110,000	0.11	0.10	0.11	0.695
$110,001 and over	0.21	0.21	0.21	0.920

Respondents were allowed to choose any combination of these race and ethnicity categories. No respondents had income of less than $50,000 by survey design. $50,001–$60,000 is the omitted income category in the regressions.

**Table 2.** Comparison of enrollment behavior across conditions (new employee sample)

Dependent variables	Full sample (*n* = 600)	*Short* condition (*n* = 302)	*Long* condition (*n* = 298)	p-value
Enrollment in 401(k) plan ( = 1 if yes)	0.90 (0.30)	0.88 (0.32)	0.92 (0.27)	0.084
Confidence in the enrollment decision	78.98 (20.44)	78.76 (21.13)	79.19 (19.75)	0.958
Contribution rate (% of salary contributed if enrolled)	6.59 (7.70)	6.69 (8.50)	6.44 (6.85)	0.863
Default allocation (fraction choosing default of 50% stocks and 50% bonds)	0.61 (0.49)	0.61 (0.49)	0.61 (0.49)	0.980
Asset allocation (% of contribution allocated to stocks if not default)	58.57 (20.02)	57.73 (21.12)	59.39 (18.95)	0.613

**Table 3.** Estimated marginal effects on enrollment behavior (new employee sample)

Explanatory variables	Dependent variable				
	Enrollment in 401(k) plan	Confidence of enrollment decision	Contribution rate (if enrolled)	Default allocation (if enrolled)	% Stocks chosen (if not the default)
Short form (=1 if yes)	−0.037 (0.023)	−0.210 (1.598)	0.175 (0.669)	0.003 (0.039)	−1.300 (2.825)
Financial literacy (# of questions correct; ranges from 0 to 5)	0.005 (0.010)	3.136*** (0.707)	−0.019 (0.297)	−0.087*** (0.016)	3.421** (1.452)
Part-time worker (=1 if yes)	−0.143** (0.065)	−6.953** (2.970)	1.368 (1.336)	0.171** (0.076)	−9.846 (8.619)
Age	0.003* (0.001)	0.300*** (0.091)	0.049 (0.039)	−0.009*** (0.002)	−0.224 (0.145)
Male ( = 1 if male)	0.015 (0.024)	−1.085 (1.627)	−0.316 (0.684)	0.073* (0.040)	−2.547 (2.897)
Married ( = 1 if yes)	0.062** (0.028)	3.658** (1.724)	−0.875 (0.725)	0.055 (0.042)	1.181 (3.116)
Race/ethnicity					
Hispanic	0.089*** (0.022)	4.107* (2.137)	0.039 (0.885)	0.011 (0.052)	2.300 (3.942)
Native American	−0.039 (0.076)	−6.470* (3.857)	−1.307 (1.674)	0.035 (0.108)	−6.317 (8.818)
Asian	−0.002 (0.054)	−3.003 (3.644)	−1.529 (1.655)	0.200** (0.094)	16.120 (11.185)
Black	−0.046 (0.059)	−2.883 (3.386)	0.510 (1.550)	0.074 (0.103)	9.662 (10.647)
Hawaiian or Pacific Islander	−0.098 (0.123)	−5.361 (5.773)	−0.427 (2.555)	−0.041 (0.176)	6.984 (14.171)
White	−0.055 (0.034)	−6.217* (3.167)	0.654 (1.478)	0.047 (0.106)	5.486 (10.184)
Income					
$60,001–$70,000	−0.084 (0.054)	0.224 (2.659)	0.016 (1.124)	−0.047 (0.067)	1.036 (4.789)
$70,001–$80,000	−0.088 (0.057)	0.399 (2.773)	−1.414 (1.178)	−0.029 (0.069)	3.300 (5.200)
$80,001–$90,000	−0.058 (0.067)	1.028 (3.203)	0.982 (1.331)	0.046 (0.075)	5.086 (5.904)
$90,001–$100,000	−0.121 (0.076)	1.542 (3.297)	1.995 (1.405)	−0.003 (0.082)	2.334 (6.095)
$100,001–$110,000	−0.033 (0.055)	1.987 (2.944)	1.129 (1.229)	−0.075 (0.074)	7.909 (4.994)
$110,001 and over	−0.054 (0.047)	7.325*** (2.432)	1.138 (1.011)	−0.019 (0.060)	−1.331 (4.561)
*N*	600	600	541	541	209
Pseudo *R*^2^	0.12	0.13	0.03	0.11	0.09

Column 1 reports the marginal effects of a probit regression of the choice to enroll (enroll = 1) in the plan. Column 2 reports the results of an OLS regression of confidence in enrollment decision (measured on a scale from 0 to 100). Column 3 reports the result of an OLS regression of the contribution rate (as a percentage of annual salary) conditional on enrollment = 1. Column 4 reports the marginal effect of a probit regression of the binary choice of staying with the default asset allocation conditional on enrollment = 1. Column 5 presents the results of an OLS regression of the % of contribution allocated toward stocks if the default is not chosen (measured from 0 to 100). In all models, the constant term is included but not shown and standard errors are in parentheses. No respondents had income of less than $50,000 by survey design. $50,001–$60,000 is the omitted income category in the regressions.

*** Significance at the 1% level,

** significance at the 5% level,

* significance at the 10% level.

**Table 4.** Descriptive statistics by condition (seeking-employment sample)

Socioeconomic explanatory variables	Full sample (*n* = 420)	*Short* condition (*n* = 205)	*Long* condition (*n* = 215)	p-value
Financial literacy (# of questions correct; ranges from 0 to 5)	2.92	2.98	2.86	0.291
Part-time worker ( = 1 if yes)	0.29	0.35	0.24	0.024
Looking for work now ( = 1 if yes)	0.65	0.64	0.66	0.838
Age	38.46	38.14	38.76	0.832
Male ( = 1 if male)	0.25	0.28	0.22	0.175
Married ( = 1 if yes)	0.27	0.26	0.28	0.584
Race/ethnicity^2^				
Hispanic	0.11	0.10	0.13	0.358
Native American	0.04	0.04	0.05	0.495
Asian	0.07	0.07	0.07	0.896
Black	0.22	0.20	0.23	0.555
Hawaiian or Pacific Islander	0.02	0.02	0.02	0.946
White	0.71	0.071	0.071	0.744
Income level				
$0–$10,000	0.45	0.42	0.48	0.203
$10,001–$20,000	0.18	0.19	0.18	0.916
$20,001–$30,000	0.14	0.16	0.12	0.263
$30,001–$40,000	0.06	0.07	0.06	0.552
$40,001–$50,000	0.05	0.04	0.05	0.915
$50,001 +	0.12	0.12	0.11	0.879

Respondents were allowed to choose any combination of these race and ethnicity categories.

**Table 5.** Comparison of enrollment behavior across conditions (seeking-employment sample)

Dependent variables	Full sample (*n* = 420)	*Short* condition (*n* = 205)	*Long* condition (*n* = 215)	p-value
Enrollment in 401(k) plan ( = 1 if yes)	0.68 (0.47)	0.70 (0.46)	0.67 (0.46)	0.476
Confidence in the enrollment decision	66.21 (28.53)	68.72 (27.39)	64.62 (29.50)	0.266
Contribution rate (% of salary contributed if enrolled)	7.80 (11.10)	8.23 (11.30)	7.37 (10.93)	0.091
Default allocation (fraction choosing default of 50% stocks and 50% bonds)	0.64 (0.48)	0.65 (0.48)	0.64 (0.48)	0.805
Asset allocation (% of contribution allocated to stocks if not default)	52.66 (19.45)	54.54 (19.00)	50.85 (19.90)	0.182

**Table 6.** Estimated marginal effects on enrollment behavior (seeking-employment sample)

Explanatory variables	Dependent variable				
	Enrollment in 401(k) plan	Confidence of enrollment decision	Contribution rate (if enrolled)	Default allocation (if enrolled)	% stocks chosen (if not the default)
Short form (=1 if yes)	0.016 (0.044)	4.017 (2.705)	0.896 (1.349)	0.013 (0.055)	4.293 (4.257)
Financial literacy (# of questions answered correctly; ranges from 0 to 5)	0.069*** (0.019)	2.526** (1.179)	−0.005 (0.582)	−0.045* (0.024)	−0.597 (1.866)
Part-time worker (=1 if yes)	0.010 (0.055)	−1.883 (3.298)	0.124 (1.626)	0.016 (0.066)	−1.574 (5.109)
Looking for work now	0.031 (0.051)	2.884 (3.080)	0.511 (1.523)	0.024 (0.062)	0.851 (4.699)
Age	0.002 (0.002)	0.387*** (0.108)	0.106* (0.053)	−0.007*** (0.002)	−0.112 (0.168)
Male ( = 1 if male)	−0.027 (0.052)	−4.462 (3.181)	1.210 (1.600)	0.086 (0.064)	−2.149 (5.278)
Married ( = 1 if yes)	0.074 (0.051)	5.009 (3.172)	1.006 (1.523)	−0.074 (0.064)	0.744 (4.641)
Race/ethnicity					
Hispanic	−0.081 (0.073)	−3.828 (4.284)	2.583 (2.285)	0.016 (0.095)	0.154 (7.559)
Black	−0.063 (0.055)	−1.344 (3.337)	1.391 (1.773)	−0.086 (0.075)	−1.869 (5.515)
Income					
$0–$10,000	−0.125** (0.005)	−7.758 (3.157)	−0.851 (1.570)	0.013 (0.064)	1.772 (5.210)
$10,001–$20,000	−0.093 (0.067)	−3.450 (3.874)	−3.294* (1.903)	−0.005 (0.079)	−0.952 (5.761)
*N*	420	420	286	286	102
Pseudo *R*^2^	0.072	0.109	0.040	0.063	0.026

Column 1 reports the marginal effects of a probit regression of the choice to enroll (enroll = 1) in the plan. Column 2 reports the results of an OLS regression of confidence in enrollment decision (measured on a scale from 0 to 100). Column 3 reports the result of an OLS regression of the contribution rate (as a percentage of annual salary) conditional on enrollment = 1. Column 4 reports the marginal effect of a probit regression of the binary choice of staying with the default asset allocation conditional on enrollment = 1. Column 5 presents the results of an OLS regression of the % of contribution allocated toward stocks if the default is not chosen (measured from 0 to 100). In all models, the constant term is included but not shown and standard errors are in parentheses. The omitted income category in the regressions is $20,000+.

*** Significance at the 1% level,

** significance at the 5% level,

* significance at the 10% level.

**Table 7.** Descriptive statistics by condition (student sample)

Socioeconomic explanatory variables	Full sample (*n* = 233)	*Short* condition (*n* = 116)	*Long* condition (*n* = 117)	p-value
Financial literacy (# of questions answered correctly; ranges from 0 to 5)	3.87	3.96	3.79	0.136
Not employed ( = 1 if yes)	0.47	0.42	0.52	0.149
Age	22.18	22.16	22.19	0.504
Male ( = 1 if male)	0.45	0.50	0.40	0.148
Married ( = 1 if yes)	0.07	0.06	0.08	0.797
Race/ethnicity*				
Hispanic	0.17	0.16	0.18	0.726
Native American	0.04	0.03	0.04	0.990
Asian	0.05	0.06	0.04	0.570
Black	0.04	0.04	0.04	0.989
White	0.88	0.88	0.88	0.981

* Respondents were allowed to choose any combination of these race and ethnicity categories.

**Table 8.** Comparison of enrollment behavior across conditions (student sample)

Dependent variables	Full sample (*n* = 233)	*Short* condition (*n* = 116)	*Long* condition (*n* = 117)	p-value
Enrollment in 401(k) plan ( = 1 if yes)	0.94 (0.25)	0.92 (0.27)	0.95 (0.22)	0.413
Confidence in the enrollment decision	75.42 (20.71)	76.81 (19.78)	74.05 (21.59)	0.414
Contribution rate (% of salary contributed if enrolled)	6.60 (5.68)	6.86 (6.18)	6.35 (5.16)	0.584
Default allocation (fraction choosing default of 50% stocks and 50% bonds)	0.50 (0.50)	0.55 (0.50)	0.45 (0.50)	0.136
Asset allocation (% of contribution allocated to stocks if not default)	61.16 (18.35)	61.77 (18.43)	60.67 (18.42)	0.752

**Table 9.** Estimated marginal effects on enrollment behavior (student sample)

Explanatory variables	Dependent variable				
	Enrollment in 401(k) plan	Confidence of enrollment decision	Contribution rate (if enrolled)	Default allocation (if enrolled)	% stocks chosen (if not the default)
Short form (=1 if yes)	−0.022 (0.032)	1.784 (2.613)	0.473 (0.785)	0.121* (0.066)	0.496 (3.618)
Financial literacy (# of questions correct; ranges from 0 to 5)	0.008 (0.016)	3.950*** (1.361)	0.728* (0.408)	−0.022 (0.034)	5.155*** (1.871)
Not employed (=1 if yes)	−0.014 (0.032)	−2.246 (2.607)	−0.361 (0.783)	0.088 (0.066)	−0.080 (3.601)
Age	0.005 (0.007)	0.990*** (0.334)	0.041 (0.098)	−0.009 (0.009)	−0.458 (0.352)
Male ( = 1 if male)	−0.033 (0.033)	3.095 (2.670)	−0.389 (0.803)	−0.107 (0.067)	3.899 (3.570)
Race/ethnicity					
Hispanic	−0.033 (0.052)	−3.594 (3.723)	1.771 (1.123)	0.126 (0.094)	−2.184 (5.615)
Native American	−0.293 (0.292)	−13.233 (12.642)	−2.753 (4.318)	−0.112 (0.380)	11.745 (18.538)
Asian	−0.388 (0.306)	−22.882* (12.563)	−1.365 (4.354)	0.122 (0.387)	32.374 (26.408)
Black	−0.168 (0.236)	−14.903 (12.644)	−1.740 (4.799)	−0.361 (0.271)	19.117 (23.977)
White	−0.049 (0.059)	−10.163 (12.075)	−1.143 (4.365)	−0.170 (0.365)	17.745 (22.840)
*N*	233	233	218	218	109
Pseudo *R*^2^	0.106	0.14	0.033	0.060	0.022

Column 1 reports the marginal effects of a probit regression of the choice to enroll (enroll = 1) in the plan. Column 2 reports the results of an OLS regression of confidence in enrollment decision (measured on a scale from 0 to 100). Column 3 reports the result of an OLS regression of the contribution rate (as a percentage of annual salary) conditional on enrollment = 1. Column 4 reports the marginal effect of a probit regression of the binary choice of staying with the default asset allocation conditional on enrollment = 1. Column 5 presents the results of an OLS regression of the % of contribution allocated toward stocks if the default is not chosen (measured from 0 to 100). In all models, the constant term is included but not shown and standard errors are in parentheses.

*** Significance at the 1% level,

** significance at the 5% level,

* significance at the 10% level.

## Footnotes

1 Wiener and Doescher (2008), Benartzi and Thaler (2013), Siedle (2013), Miller et al. (2015), Vernon (2015), and Kirkham (2016) show similar numbers.

2 A 401(k) plan is a tax deferred, defined contribution, retirement savings account. These plans are typically setup by employers and offered to employees as a means to encourage retirement savings. Employees who opt-in, make contribution via payroll withholdings, pre-tax, and these contributions can be matched (partially) by the employer. The employee then pays income tax when they withdraw money from this account, typically during retirement. This type of plan is named after a section of IRS code, and is prevalent in the private sector in the US.

3 Similarly, Bhargava and Manoli (2015) find that reducing information complexity can increase the proportion of eligible people who claim EITC benefits.

4 As a way to improve retirement-planning decisions, both academic researchers and policy makers have supported the use of nudges. Moreover, the growing research interest aimed at examining how, and the degree to which, non-price nudges can impact behaviour, spans well beyond retirement planning to a host of other domains including financial planning, education, health care, risky behaviours, consumption, and energy conservation. See Johnson et al. (2012), Madrian (2014), Benartzi et al. (2017), Loewenstein and Chater (2017), and Loewenstein et al. (2017) for discussions and reviews of this literature.

5 Some might consider this manipulation to fall under what has been referred to in the literature as a ‘signpost’ or a ‘boost’. We refer readers to Camilleri et al. (2019) for a discussion of their proposed distinction between nudges and signposts. We are less concerned about the specific nomenclature of this type of manipulation.

6 Qualtrics is an independent, third-party survey company. Via their Panels service, Qualtrics solicits a diverse sample of responses from within the US, conditional on the inclusion criteria set forth by the survey administrators. Qualtrics charges a flat-rate per completed survey, and respondents are then compensated directly by Qualtrics.

7 Because this study focuses on planning decisions with regard to 401(k) plans, we focus our attention primarily to the prior literature that examines enrollment, contribution, and investment choices related to 401(k) plans or equivalent defined-contribution plans.

8 However, Beshears et al. (2017) provide robust evidence that providing information about returns does not impact portfolio allocations.

9 As Bassett et al. (1998) and Benartzi and Thaler (2007) point out, participating in a 401(k) plan shifts the responsibility of saving and investing for retirement to the employee and, as the cost of making financial mistakes can be enormous (Campbell, 2006), individuals with low financial knowledge may prefer to stay away from participating in 401(k) plans (Brown & Weisbenner, 2014; Campbell, 2006).

10 More generally, the link between financial literacy and improved retirement planning has also been well documented across different types of programs and countries. We refer readers to Lusardi & Mitchell (2014) for a comprehensive review and discussion.

11 The authors attribute this to the fact that married individuals, unlike single adults, have access to more than one source of employment income, making them potentially aggressive in their 401(k) allocations. Another possible reason is that being married is more closely aligned with intergenerational wealth transfers and hence a stronger desire to invest in long-term assets.

12 The five financial literacy questions were the following: (1) Suppose you have $100 in a savings account earning 2% interest a year. After 5 years, how much would you have? (More than $102, Exactly $102, Less than $102), (2) Imagine that the interest rate on your savings account is 1% a year and inflation is 2% a year. After one year, would the money in the account buy more than it does today, exactly the same or less than today? (More, Same, Less), (3) If interest rates rise, what will typically happen to bond prices? (Rise, Fall, Stay the same, No relationship), (4) True or False: A 15-year mortgage typically requires higher monthly payments than a 30-year mortgage but the total interest over the life of the loan will be less. (True, False), (5) True or False: Buying a single company’s stock usually provides a safer return than a stock mutual fund. (True, False). Because the financial literacy questions were asked prior to the actual experimental task, there are some potential priming affects. From the perspective of the respondents, we thought this was the most natural place in the survey flow for these questions – lumped in with the other background questions on employment status and other social-demographic questions. By asking these financial literacy questions first, we thought this could have the (indirect) effect of getting respondents engaged and, thus, increasing the focus and intentionality or respondents in the experimental task. That said, we are cautious in how we interpret results relating to this measure of financial literacy; in particular, we do not claim any causal inferences and, instead, interpret these findings as correlational between financial literacy and our dependent choice variables related to planning behaviors.

13 For brevity, we refer to these hypothetical choices in this choice experiment as anticipated behaviors/choices throughout analysis of the result and drop the repeated use of the word hypothetical, as it is henceforth implied.

14 As part of the experimental design planning, research assistants compiled publically available information about employer-sponsored, retirement plans from both large corporations and universities. The hypothetical plan information in the study was modeled from these real-world templates. As an illustrative example, we include a link to the publically available plan description provided by Texas Tech University, where the authors are affiliated.

15 Importantly, this 73% self-reported participation rate is consistent with the national participation rates reported in a recent ICI Research Perspective (Brady & Bass, 2019) of high-income individuals. Based on 2016 IRS fillings, 78% of individuals with annual incomes between $50,000 and $100,000 participate in a retirement plan, while 85% of individuals with annual income over $100,000 participate.

16 Qualtrics complete their own series of quantity control checks on the collected sample. In particular, they incorporate a primary ‘speeding’ check filter that removes respondents who are deemed to be going too fast through the study (calibrated based on duration results from a pilot sample of the same survey). In our case, this filter was set at just over one minute. Thus, no one in our sample spend less than 1 minute completing the survey. But the mean of 6 minutes (and median of 4 minutes) suggest that a majority of respondents spent a sufficient amount of time completing the survey.

17 Importantly, we observe these high rates of anticipated enrollment even though we offer an explicit choice to ‘wait to enroll at a later time’. Consequently, this gives us more confidence that the *explicit* enrollment choice in the task reflects preferences regarding anticipated behavior; in particular, if they had some indecision or uncertainty, they had an opportunity to choose to *wait*, but this option was rarely chosen.

18 We also examined the enrollment decision via a Probit model that included a dummy variable indicating whether the participant participated in an employer-sponsored plan in real life as an additional regressor. This did not change the result that the treatment had no effect on enrollment, as reported in the Supplemental Appendix.

19 We acknowledge that the high level of reported, anticipated participation does make it more difficult to identify possible increases in participation resulting from the Short condition. That said, the point estimates are actually higher for the Long condition compared to the Short condition, which is opposite to our hypothesized direction; thus the fact that we don’t observe significantly more participation in the Short condition is likely not a ‘ceiling-effect’ problem. Moreover, by way of foreshadowing future results, we show in Section 5 the same general result for a subsample of respondents with much lower anticipated participation rates, where a possible ceiling-effect is not a concern.

20 We also examined an alternative model that included the percentage of salary the experimental participant contributed in an employer-sponsored plan in real life as an additional regressor. This did not change the result that the treatment had no effect on contribution level, as reported in the Supplemental Appendix.

21 In practice the portfolio allocation decision would be much more complex and likely involve choosing between a menu of stock-funds and bond-funds (or other fixed income funds). In this regard, we are not purporting that this stylized asset allocation is perfectly representative. Rather, our intent was to operationalize the portfolio allocation in this easy-to-understand way to respondents, as a way to proxy for the overall portfolio distribution between stocks and bonds.

22 Interestingly, we see lower anticipated enrollment among the seeking-employment sample (68%) compared to the employed sample (90%); however, this is likely attributed to the fact that a much lower fraction of the seeking-employment sample had answered affirmatively to having ever participated in an employer-sponsored plan (42% compared to 73%). Hence, we expect much less anchoring from actual experience, which is the intended purpose of this additional sample. However, conditional on enrollment, the remaining choices exhibit a similar pattern to that of the employed sample. In addition, we also see slightly higher average anticipated contribution rates among the seeking-employment sample (7.80) compared to the employed sample (6.59) and student sample (6.60). However this small difference in anticipated contributions rates across the three samples is not statistically significant (ANOVA: *p* = 0.121).

23 Specifically, these students were enrolled in a voluntary, research participation pool. Both undergraduate and graduate students are eligible to participate, although in practice the pool is heavily weighted toward undergraduates.

24 For the sake of maintaining a consistent procedure across all conditions, we also asked respondents from this sample the same questions about anticipated retirement savings behavior and actual retirement savings behavior after the multiple-choice, plan-comprehension questions. However, given the potential that these questions could influence the responses to the subsequent retirement-planning questions, we exclude these data from the analysis.

25 We also examined the correlation between time spent of the plan info and measured financial literacy. Importantly, we found very little correlation. Specifically, in the *Long* condition there is essentially no correlation between time spent on the plan info and financial literacy (*corr* = 0.009; *p* = 0.934), while in the *Short* condition there is a modest positive correlation that is marginally significant (*corr* = 0.177; *p* = 0.079).

26 There might also be a difference in how information complexity interacts with peoples’ likelihood of actually consuming the messages. Namely, it might be that less complex messages are more likely to actually be consumed and, thus, more effective at motivating retirement planning behavior. Based on our findings, an interesting question for future research might be how varying the complexity within these follow-up messages impacts their effectiveness.

27 We also examined possible interaction effects between financial literacy and our main short-form manipulation. We found no statistically significant interaction effects on any of our outcome variables, as reported in the Supplemental Appendix. Thus, there does not appear to be a differential impact of financial literacy.

28 It is beyond the scope of this paper to go into the specifics of how to optimally design defaults, but we view this as an important area for future research. In particular, a reviewer aptly pointed out that an important consideration is how defaults are structured under a heterogeneous pool of eligible participants such as ours.
