# Supplementary material for: Less is not more: 401(k) plan information and retirement planning choices
Source: J Pension Econ Financ. Author manuscript; Available in PMC 2023 Dec 8. (PMC10704390; doi:10.1017/s1474747221000445)
Supplement: Sup material [file NIHMS1909809-supplement-Sup_material.docx]

**Supplemental Appendix A: Copy of Experimental Instructions**

**Instructions Prior to Receiving Plan Info and Retirement Questions**

This survey has several parts. In the first part of the survey, we will ask you some hypothetical questions about what decisions you would make regarding an employer-sponsored retirement account.

For this part of the survey, please imagine that you recently started a new job at a reputable company at the current salary you have now. Your new employer offers a retirement plan, which you are eligible to participate in if you choose. On the next page we will be providing you with some hypothetical information about the retirement plan that is representative of the type of information that you would actually receive from your employer at the time you start the job. After reading through the information about the plan, we are going to ask you some questions about whether you would enroll in the plan, how much you would contribute to the plan if you enrolled, and how you would invest your contributions.

We kindly ask that you carefully consider all the information presented, that you seriously consider the options available, and that you answer honestly based on what decisions you think you would make given the specific information that is provided about the retirement plan. When thinking about what retirement planning decisions you would make for this study, please answer the questions based on what you would do based on your current life circumstances.

***Long* Version Condition: 401(k) Plan Information**

***Overview***

As a condition of your employment, you are eligible to save for retirement through our optional, employer-sponsored, 401(k) plan. The 401(k) plan is an individualized defined-contribution plan with employer matching to which you will contribute a percentage of your salary each paycheck. If you participate, you will manage your own personal account and choose from a variety of different investment options that are offered through a company-authorized investment firm. Retirement benefits from participation in the 401(k) are a direct result of any amount contributed by you, the amount we, as your employer, contribute, and any returns you earn on the investments you select.

***Enrollment***

If you desire to enroll in the plan, you will have 30 days from your date of hire to enroll, to choose what percentage of your salary you wish to contribute, and to make your investment choices. If you do not elect to enroll in the 30 day period, you will not be enrolled. If you do enroll but do not elect a contribution percentage, the default of 4% will be chosen for you. Changes to your enrollment status, your contribution percentage, and your investment choices can be made once a year during the open enrollment period. It is generally regarded as a good idea to contribute to an employer-sponsored 401(k) plan when there is matching. You contribution and the addition of the employer match increases the amount you are able to save for retirement.

***Investment Options***

If you choose to enroll, you also will need to choose how to allocate these contributions. Your investment options include several mutual funds. A mutual fund is a collection of stocks and/or bonds. You, as an investor, purchase shares in this fund, and thus own a much more diversified (less risky) portfolio than you would if you purchased individual stocks or bonds. Stocks provide a higher rate of return than bonds on average but are riskier to hold. Bonds generally are considered less risky than stocks but offer a lower rate of return on average. The younger you are, the more risk you can afford to take, and the greater the percentage of stocks your investment portfolio should have. As you get older, you should reduce the percentage of stocks in your portfolio and increase the percentage of bonds. A general rule of thumb is that the percentage of your portfolio invested in bonds, bond funds, or other fixed-income securities should equal your age. If you enroll in the plan, but do not choose how to allocate your contribution across the different investment options, your contributions will be automatically invested 50% in stocks and 50% in bonds.

***Matching***

We, as your employer, will provide a 100% match to any funds that you contribute to the 401(k) plan up to 4% of your salary. For example, if you contribute 3% of your salary, we will contribute another 3% of your salary. If you contribute 4% of your salary, we will contribute 4%. If you contribute 5% of your salary, we will contribute 4% of your salary. The IRS contribution limit for employees is $18,000, and the total employer plus employee contribution limit is $54,000 for 2017. However, if you are aged 50 or older, you are eligible to contribute an additional $6000 above the $18,000 yearly limit, for a maximum contribution of $24,000 in 2017. In order to take full advantage of the employer match, you should contribute 4 percent, but a general rule of thumb is that you should be saving at least 10 percent of your income toward retirement.

***Vesting***

Your contributions and the earnings on your contributions are yours from your first day of employment. However, you must be employed with us at least 3 years to keep the employer contributions and the earnings on those contributions. If you are employed with us for less than 3 years you will forfeit the employer portion of contributions and earnings.

***Distribution***

In general, you are not able to withdraw the money that has accrued in your 401(k) requirement account until one of the following occurs:

1. You die, become disabled, or otherwise discontinue your employment with us.
2. This 401(k) plan is terminated and is not replaced by a successor plan.
3. You reach age 59 ½ or incur a financial hardship.

You will pay income taxes on any distributions from the plan unless you roll over your distribution to another 401(k) or IRA. In addition, if you are under the age of 59 ½ at the time of the distribution, any portion not rolled over may be subject to a 10% penalty.

***Tax Benefits***

In addition to preparing for retirement, contributing to a 401(k) plan has the added benefit of reducing your current federal income taxes because contributions are not subject to income tax at the time they are made. In addition, the earnings on these contributions are tax-deferred, meaning that you will not pay income taxes on the funds in your 401(k) account until they are withdrawn or paid out in retirement.

***Disclaimer***

Investment earnings are not guaranteed. Therefore, you should consider your enrollment, contribution percentage, and investment choices carefully. Factors to consider include your age, your current and future salary, how long you expect to be employed with us, your retirement age, your participation in other retirement plans, your risk tolerance, and your ability to manage your own investments.

***Short* Version Condition: 401(k) Plan Information**

You are eligible to save for retirement through our optional, employer-sponsored, 401(k) plan. The table below provides some important information about this optional 401(k) plan:

|  | **Overview of Important Plan Information** |
| --- | --- |
| **Type of Plan** | - **401(k) plan with employer matching** - **We, as your employer, will contribute a dollar into your 401(k) account for every dollar you contribute, up to 4% of your salary** |
| **Enrollment** | - **You have 30 days to enroll and choose a % of your salary to contribute** |
| **Investment Options** | - **You choose what % of your salary you wish to contribute to the plan** - **You choose the percentages to be invested in stock and bond mutual funds** |
| **Vesting** | - **All of the money your contribute and the earnings from your contributions are automatically yours to keep from the time you enroll in the plan** - **The matching money from your employer may only be kept once you’ve completed 3 years of service** |
| **Distribution** | - **In general, you will not be able to withdraw money from the plan until you retire or are 59 ½ years old** - **Early withdrawals will be assessed a 10% penalty** |
| **Tax Benefits** | - **Contributions to the plan are tax deferred which reduces your current income taxes** - **You do not have to pay income tax on the money in the plan until it is withdrawn** |
| **Defaults** | - **If you do not do anything within 30 days, you will not be enrolled** - **If you enroll but don’t make any contribution decisions, your contribution level will automatically be set to 4%** - **If you enroll but don’t make any investment decisions, your contribution and the employer match will be invested 50% in bond funds and 50% in stock funds** |

Below are some useful guidelines to help you with your enrollment and contribution decisions:

***Should I enroll?***

Yes, it is generally regarded as a good idea to contribute to an employer-sponsored, 401(k) plan when there is matching. Your contribution and the addition of the employer match increases the amount you are able to save for retirement.

***How much should I contribute?***

In order to take full advantage of the employer match, you should contribute 4%, but a general rule of thumb is that you should be saving at least 10% of your income toward retirement.

***How should I invest my contribution?***

A general rule of thumb is that the amount invested in bonds is equal to your age.

***Disclaimer***

Investment earnings are not guaranteed. Therefore, you should consider your enrollment, contribution percentage, and investment choices carefully. Factors to consider include your age, your current and future salary, how long you expect to be employed with us, your retirement age, your participation in other retirement plans, your risk tolerance, and your ability to manage your own investments.

**Supplemental Appendix B: Additional Results from Alternative Specifications**

Below we present the results from series of robustness checks using alternative samples that are referenced in the main paper. As you will see and consistent with the result from our main specifications reported in the main text, there are no statistically significant effects of the short form on enrollment or contribution rates.

|  | **Dependent Variable** | | | | |
| --- | --- | --- | --- | --- | --- |
| ***Explanatory Variables*** | ***Enrollment in 401(K) Plan*** | ***Enrollment in 401(K) Plan*** | ***Contribution Rate***  ***(if enrolled)*** | ***Contribution Rate***  ***(if enrolled)*** |  |
| *Short Form* (= 1 if yes) | -0.023  (0.023) | -0.037  (0.024) | 0.379  (0.747) | 4.151  (2.868) |  |
| *Financial Literacy* (# of questions correct; ranges from 0 to 5) | 0.001  (0.009) | 0.006  (0.010) | 1.099***  (0.352) | 0.021  (0.435) |  |
| *Real Life Participation (=1 if yes)* | 0.079**  (0.037) |  |  |  |  |
| *Real Life Contribution* |  |  | 0.216***  (0.027) |  |  |
| *Short Form* X *Financial Literacy* |  | Yes^a^ |  | -0.058  (0.590) |  |
| *Short Form X Age* |  | Yes^b^ |  | -0.109  (0.072) |  |
| *Part-time worker* (= 1 if yes) | 0.006  (0.046) | -0.141**  (0.065) | 3.659**  (1.692) | 1.434  (1.339) |  |
| *Age* | 0.004**  (0.002) | 0.003*  (0.001) | 0.062  (0.044) | 0.099*  (0.051) |  |
| *Male* (= 1 if male) | 0.034  (0.023) | 0.015  (0.024) | -0.758  (0.773) | -0.358  (0.684) |  |
| *Married* (=1 if yes) | 0.021  (0.026) | 0.063**  (0.028) | -0.898  (0.812) | -0.888  (0.726) |  |
| *Race/ethnicity dummies* | Yes | Yes | Yes | Yes |  |
| *Income dummies* | Yes | Yes | Yes | Yes |  |
| N | 526 | 600 | 410 | 541 |  |
| (Pseudo) R^2^ | 0.13 | 0.12 | 0.187 | 0.040 |  |
| **Notes:** Columns 1 & 2 report the marginal effects of a probit regression of the choice to enroll (enroll =1) in the plan. Columns 3 & 4 report the result of an OLS regression of the contribution rate (as a percentage of annual salary) conditional on enrollment = 1.  ^a^ Interaction term was included in this specification and was not statistically significant  ^b^ Interaction term was included in this specification and was not statistically significant  ***Significance at the 1% level, **Significance at the 5% level, *Significance at the 10% level. | | | | | |
